# Can FDG-PET/CT replace blind bone marrow biopsy of the posterior iliac crest in Ewing sarcoma?

**DOI:** 10.1007/s00256-017-2807-2

**Published:** 2017-11-09

**Authors:** Ömer Kasalak, Andor W. J. M. Glaudemans, Jelle Overbosch, Paul C. Jutte, Thomas C. Kwee

**Affiliations:** 1 Department of Radiology, Nuclear Medicine and Molecular Imaging, University Medical Center Groningen, University of Groningen, Hanzeplein 1, PO Box 30.001, 9700 RB Groningen, The Netherlands; 2 Department of Orthopedics, University Medical Center Groningen, University of Groningen, Groningen, The Netherlands

**Keywords:** Biopsy, Bone, Ewing sarcoma, Pet-Ct, Tumor staging

## Abstract

**Objective:**

To determine and compare the value of ^18^F–fluoro-2-deoxy-d-glucose positron emission tomography/computed tomography (FDG-PET/CT) to blind bone marrow biopsy (BMB) of the posterior iliac crest in detecting metastatic bone marrow involvement in newly diagnosed Ewing sarcoma.

**Materials and methods:**

This retrospective study included 20 patients with newly diagnosed Ewing sarcoma who underwent pretreatment FDG-PET/CT and a total of 38 blind BMBs (two unilateral and 18 bilateral) of the posterior iliac crest. FDG-PET/CT scans were evaluated for bone marrow involvement, both in the posterior iliac crest and other sites, and compared to blind BMB results.

**Results:**

FDG-PET/CT was positive for bone marrow involvement in 7/38 posterior iliac crests, whereas BMB was positive in 5/38 posterior iliac crests. FDG-PET/CT and BMB results in the posterior iliac crest agreed in 36/38 cases (94.7%, 95% confidence interval [CI]: 82.7–98.5%). On a patient level, FDG-PET/CT was positive for bone marrow involvement in 4/20 patients, whereas BMB of the posterior iliac crest was positive in 3/20 patients. On a patient level, FDG-PET/CT and BMB results agreed in 19/20 patients (95.0%, 95% CI: 76.4–99.1%). The only discrepancies between FDG-PET/CT and BMB were observed in two BMBs of one patient. Both BMBs in this patient were negative, whereas FDG-PET/CT indicated bilateral posterior iliac crest involvement and also extensive bone marrow involvement elsewhere.

**Conclusions:**

FDG-PET/CT appears to be a valuable method for metastatic bone marrow assessment in newly diagnosed Ewing sarcoma. The routine use of blind BMB of the posterior iliac crest should be reconsidered when FDG-PET/CT is available.

## Introduction

Ewing sarcoma is an aggressive primary tumor of bone and soft tissue occurring at any age with a peak incidence in adolescents and young adults [1], and an overall incidence of approximately 2.93 cases/1,000,000 [2]. Approximately 26–28% of newly diagnosed Ewing sarcoma patients have been reported to present with distant metastases, with lung and bone marrow being the most frequent sites of metastatic disease [2]. Metastatic status at diagnosis is the strongest prognostic factor across different treatment strategies [1]. Five-year overall survival is approximately 30% for patients with initially metastatic disease [1]. Furthermore, metastatic status is the most important factor in the risk-adapted treatment strategy that is currently employed in both North America and Europe [1]. Accurate staging is thus crucial because of its prognostic and therapeutic implications.

Patients with newly diagnosed Ewing sarcoma usually undergo conventional radiographic and magnetic resonance imaging (MRI) examinations of the primary tumor site, chest computed tomography (CT), whole-body bone scintigraphy, and blind bone marrow biopsy (BMB) of the posterior iliac crest (usually bilateral). BMB, however, is an invasive procedure that carries a small but nonnegligible risk of (hemorrhagic) complications [3], and is often performed under general anesthesia in this mainly pediatric population. Meanwhile, ^18^F–fluoro-2-deoxy-d-glucose (FDG) positron emission tomography (PET)/CT is increasingly used as a new staging method in Ewing sarcoma [4]. The exact role of FDG-PET/CT and its value compared to the traditionally used staging procedures is still unclear. However, one of the advantages of FDG-PET/CT is that it can visualize the entire body, including the bone marrow. It can be hypothesized that FDG-PET/CT may replace blind BMB of the posterior iliac crest if it is sufficiently accurate to detect bone marrow involvement.

The purpose of this study was therefore to determine and compare the diagnostic value of FDG-PET/CT to blind BMB of the posterior iliac crest in detecting metastatic bone marrow involvement in newly diagnosed Ewing sarcoma.

## Materials and methods

### Study design and patients

The local institutional review board approved this retrospective study and waived the requirement for informed consent. The database of our tertiary university hospital, which is also a referral center for sarcoma patients, was searched for all patients who were newly diagnosed with Ewing sarcoma and who underwent pretreatment FDG-PET/CT and BMB between August 2009 and August 2017. Inclusion criteria for this study were: newly diagnosed and histologically proven Ewing sarcoma, and availability of pretreatment FDG-PET/CT and blind BMB (either bilateral or unilateral) of the posterior iliac crest. Exclusion criteria for this study were: previously treated Ewing sarcoma and time interval of more than 30 days between FDG-PET/CT and BMB.

### FDG-PET/CT acquisition and evaluation

FDG-PET/CT was performed using an integrated PET/CT system (Biograph mCT PET/CT, Siemens, Knoxville, TN, USA) that was EANM/EARL (European Association of Nuclear Medicine/ResEARch 4 Life) accredited [5]. All patients fasted for at least 6 h before 3 MBq FDG/kg body weight was injected intravenously. Blood glucose levels were checked to be less than 11 mmol/l before FDG injection. PET/CT scanning was performed from cranial vertex to midthighs approximately 60 min after FDG administration, with 3 min per bed position. In patients with primary tumors in the lower extremities, a true whole-body FDG-PET/CT (from cranial vertex to toes) was performed. Low-dose CT with 100 kVp and 30 mAs was performed for attenuation correction and anatomical correlation. Data reconstruction was performed according to EANM guidelines [5]. FDG-PET/CT scans were evaluated by a board-certified nuclear medicine physician (A.W.J.M.G.) who knew the diagnosis and primary location of Ewing sarcoma in each patient, but was blinded to BMB results and other imaging findings. Both posterior iliac crests were assessed for bone marrow involvement, which was defined as focal FDG uptake exceeding background bone marrow FDG uptake (with or without lytic or sclerotic changes on corresponding low-dose CT) or non-FDG-avid lytic or sclerotic bone changes on low-dose CT. Other parts of the skeleton (outside of the primary tumor location) and extraskeletal sites were also assessed for metastatic disease.

### BMB acquisition and interpretation

Unilateral or bilateral blind BMBs of the posterior iliac crest were performed by (pediatric) oncologists. Younger patients underwent general anesthesia, conscious sedation was applied in older children, and adults only underwent local anesthesia. BMB samples were interpreted for bone marrow involvement by one of the attending expert bone pathologists who was blinded to FDG-PET/CT results.

### Data analysis

Percentage of agreement between FDG-PET/CT and BMB in detecting bone marrow involvement in the posterior iliac crest was calculated, along with 95% confidence intervals (CIs). Percentage of agreement between FDG-PET/CT and BMB on a patient level with regard to the detection of bone marrow involvement anywhere in the body (i.e., also considering bone marrow lesions outside of the posterior iliac crest detected at FDG-PET/CT) was also calculated, along with 95% CIs.

## Results

### Patients

A total of 22 patients were potentially eligible for inclusion. One of these patients was excluded because BMB was not performed, and one patient was excluded because the time interval between FDG-PET/CT and BMB was 115 days. Thus, 20 patients (12 males and eight females, with mean age of 15.9 ± 13.1 years [range, 5–57 years]) were finally included. Seventeen patients had primary bone Ewing sarcoma (pelvis [*n* = 5], femur [*n* = 2], humerus [*n* = 2], rib/chest wall [*n* = 2], sacrum [*n* = 2], clavicle [*n* = 1], lumbar vertebra [*n* = 1], temporal bone [*n* = 1], and tibia [*n* = 1]), whereas three patients had primary extraosseous Ewing sarcoma (soft tissues around the knee [*n* = 1], femur [*n* = 1], and intradural cervical spine [*n* = 1]). Eighteen patients underwent bilateral blind BMB and two patients underwent unilateral blind BMB of the posterior iliac crest, yielding a total of 38 BMBs. In 14 patients, FDG-PET/CT was performed before BMB, and in six patients FDG-PET/CT was performed after BMB. Mean time interval between FDG-PET and BMB was 6.0 ± 6.3 days (range, 0–22 days). Fifteen patients had localized disease, whereas five patients had metastatic disease according to FDG-PET/CT (lymph node metastases [*n* = 1], lymph node and lung metastases [*n* = 1]), lymph node and bone marrow metastases [*n* = 1], lymph node, lung and bone marrow metastases [*n* = 1], and lymph node, lung, liver, pancreatic and bone marrow metastases [*n* = 1]).

### FDG-PET/CT vs. BMB

FDG-PET/CT was positive for bone marrow involvement in seven posterior iliac crests and negative for bone marrow involvement in 31 posterior iliac crests, whereas BMB was positive in five and negative in 33 posterior iliac crests (Table 1). FDG-PET/CT and BMB results in the posterior iliac crest were in agreement in 36/38 cases (94.7%, 95% CI: 82.7–98.5%).

**Table 1:** Cross-tabulation of FDG-PET vs. BMB results for bone marrow involvement in the posterior iliac crest for a total of 38 BMBs

	BMB positive	BMB negative
FDG-PET/CT positive	5	2
FDG-PET/CT negative	0	31

On a patient level, FDG-PET/CT was positive for metastatic bone marrow involvement in four patients (in three of these patients FDG-PET/CT also showed bone marrow lesions outside the posterior iliac crests) and negative for metastatic bone marrow involvement in 16 patients, whereas BMB of the posterior iliac crest was positive in three and negative in 20 patients (Table 2). On a patient level, FDG-PET/CT and BMB results were in agreement in 19/20 patients (95.0%, 95% CI: 76.4–99.1%).

**Table 2:** Cross-tabulation of FDG-PET vs. BMB results for bone marrow involvement on a patient level for a total of 20 patients

	BMB positive	BMB negative
FDG-PET/CT positive	3	1
FDG-PET/CT negative	0	16

**Fig. 1:**
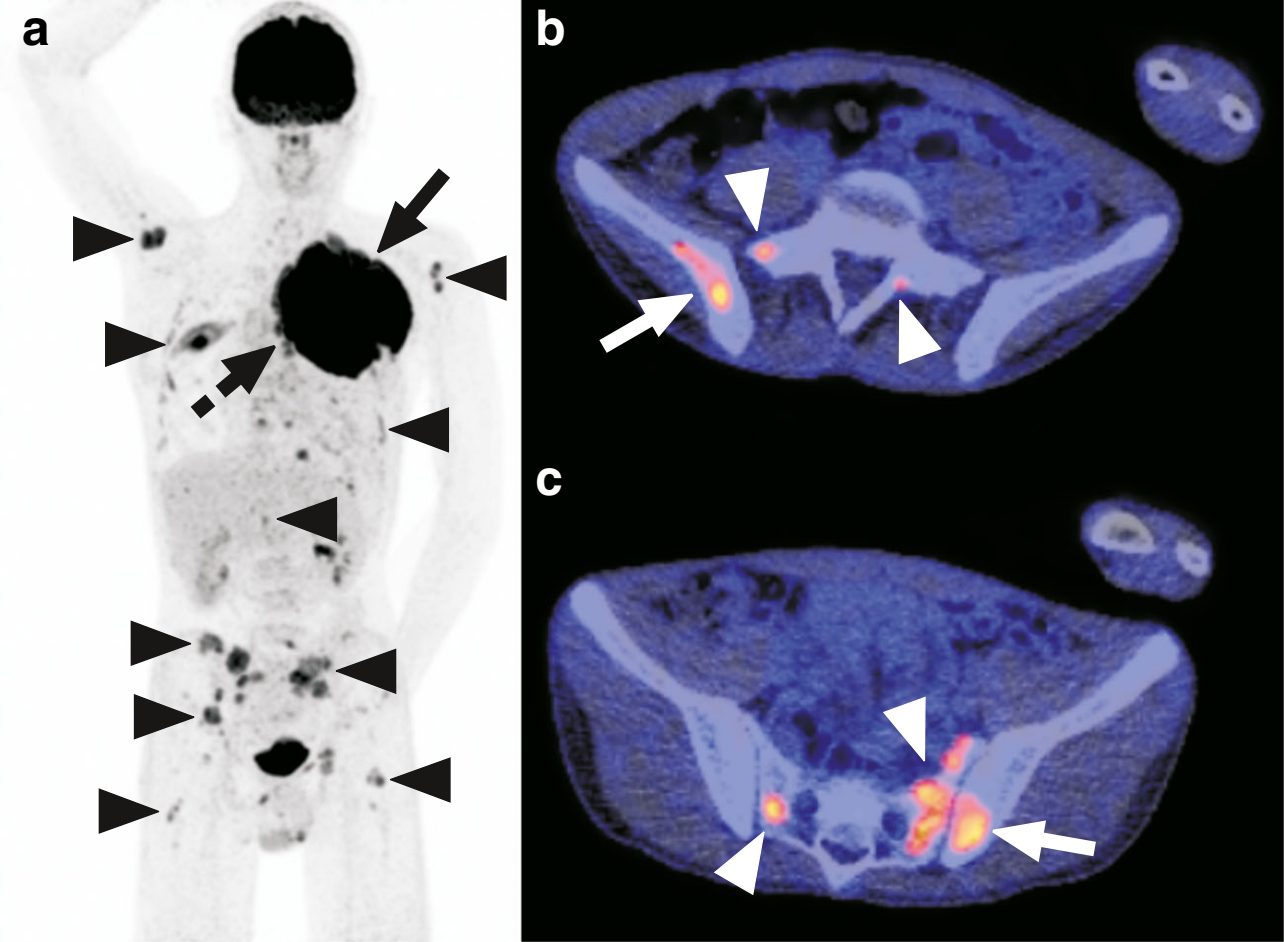
Disagreement between FDG-PET/CT and blind BMB of the posterior iliac crest in a 15-year-old boy with Ewing sarcoma. Coronal maximum intensity projection FDG-PET (**a**) shows the primary tumor in the left chest wall (*continuous arrow*), adjacent mediastinal lymph node metastases (*dashed arrow*), and extensive bone marrow metastases in both humeri, multiple ribs, vertebrae, sacrum, pelvic bone, and both proximal femora (*arrowheads*). Axial FDG-PET/CT images (**b**,** c**) show increased FDG uptake in both posterior iliac crests (*continuous arrows*) and several FDG-avid foci in the sacrum (*arrowheads*), in keeping with bone marrow metastases. Note that both posterior iliac crests are not entirely involved according to FDG-PET/CT (no increased FDG uptake is seen in the left posterior iliac crest in** b**, and no increased FDG uptake is seen in the right posterior iliac crest in** c**. Blind BMB of both posterior iliac crests showed normal maturing trilineage hematopoiesis without any malignant cells. This patient was not re-biopsied to histologically confirm bone marrow metastatic disease

The only discrepancies between FDG-PET/CT and BMB were observed in two BMBs of one patient. Both BMBs in this patient were negative, whereas FDG-PET/CT suggested bilateral posterior iliac crest bone marrow involvement and also extensive bone marrow involvement elsewhere in the axial skeleton, and both humeri and femora (Fig. 1).

**Fig. 2:**
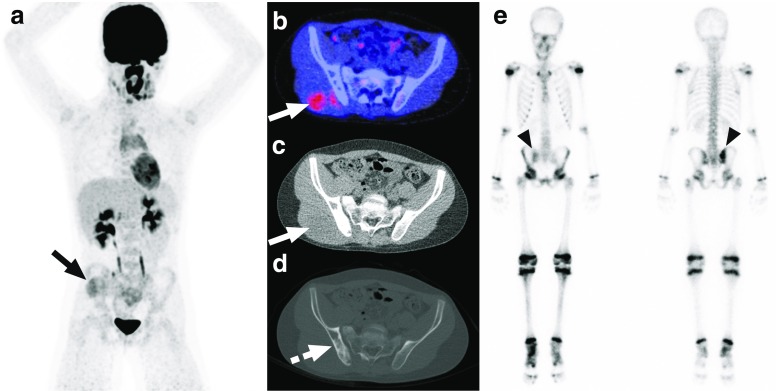
Non-FDG-avid sclerotic bone marrow involvement of the right posterior iliac crest in a 10-year-old boy with Ewing sarcoma. Coronal maximum intensity projection FDG-PET (**a**), axial FDG-PET/CT (**b**), and low-dose CT with soft tissue window settings (**c**) show the slightly FDG-avid primary tumor in the right gluteal muscles (*continuous arrow*). Although no increased FDG uptake is seen in the right posterior iliac crest (**b**), low-dose CT with bone window settings shows extensive sclerosis in the right posterior iliac crest (*dashed arrow*). Blind BMB of the right posterior iliac crest showed involvement with Ewing sarcoma. Of interest, bone scintigraphy (**e**) that was performed before FDG-PET/CT also showed pathological activity in the right posterior iliac crest (*arrowheads*)

Six FDG-PET/CT-positive posterior iliac crests exhibited focally increased FDG uptake without visible CT changes, whereas one FDG-PET/CT positive posterior iliac crest was non-FDG-avid with sclerotic changes on CT (Fig. 2).

## Discussion

The results of this study show that FDG-PET/CT and blind BMB of the posterior iliac crest are in agreement in almost all cases. The only disagreement between the two procedures in this series was observed in one patient in whom bilateral blind BMBs of the posterior iliac crest were negative, whereas FDG-PET/CT was positive at both posterior iliac crests. Although repeated CT-guided bone biopsies of FDG-avid bone foci were not performed in this patient, the fact that FDG-PET/CT showed multiple other bone lesions is highly suggestive that blind BMB was false negative in this patient due to sampling errors. Furthermore, in three other patients in whom FDG-PET/CT and blind BMB were concordantly positive at the posterior iliac crest, FDG-PET/CT detected additional bone marrow metastases outside the posterior iliac crests. Such information is important, because the number of bone lesions has been shown to correlate with a worse prognosis [1, 6]. The findings of this study thus support the hypothesis that FDG-PET/CT may replace blind BMB of the posterior iliac crest in Ewing sarcoma. On another note, FDG-PET/CT may also be used to screen for bone marrow metastases, and, if positive, subsequent CT-guided bone biopsy may be performed for histological confirmation. Given the fact that around 10% of patients have been reported to have bone marrow metastasis [7], approximately 90% of patients can be spared invasive bone biopsy with such an approach. However, this latter hypothesis was not tested in the present study.

The recently published National Comprehensive Cancer Network (NCCN) guidelines state that BMB should be considered in the workup of Ewing sarcoma, and that screening MRI of the spine/pelvis is also recommended in lieu of, or in addition to, BMB [7]. The current guidelines of the European Society for Medical Oncology (ESMO) mention that BMBs from sites distant to the primary or known metastatic lesions may be considered, in the face of a very low incidence of bone marrow metastases in localized disease, especially if PET scan was carried out [8]. Although these guidelines recognize the drawbacks of blind BMB and the potential value of imaging in this setting, ongoing studies such as the Euro Ewing 2012 trial still perform standard BMB in each patient [9]. Furthermore, the NCCN and ESMO guidelines are mostly based on expert opinions [7, 8], and actual scientific data on the value of FDG-PET/CT compared to BMB are still very scarce. One previous study by Newman et al. [10] included 69 patients with newly diagnosed Ewing sarcoma who underwent both (stand-alone) FDG-PET and (either unilateral or bilateral) BMB of the iliac crest. On a patient level, FDG-PET and BMB were positive for bone marrow metastasis in 9/69 and 6/69 patients, respectively [10]. FDG-PET suggested bone marrow metastasis in three patients who had negative BMB results, while none of the six patients with a positive BMB result were negative for bone marrow metastasis on FDG-PET [10]. These findings are completely in line with the results of the present study. To the best of our knowledge, there are no other studies that have explicitly compared FDG-PET(/CT) to BMB in Ewing sarcoma. Therefore, further research is needed to definitely establish FDG-PET/CT as a (partial) alternative to BMB.

This study had several limitations. First, the sample size was relatively small and the retrospective study design may have introduced selection bias. Future larger prospective studies are thus necessary. On another note, the results of this study may also be used by a future meta-analysis on this topic. Second, FDG-PET/CT was performed from cranial vertex to midthighs in patients with non-lower extremity primary tumors, and the region caudal to the midthighs was thus not assessed in these patients. Furthermore, although low-dose CT was performed in this study with (mainly) pediatric patients, a full-dose CT may be considered instead, since the CT component is a stand alone for the definition of a positive FDG-PET/CT. Third, FDG PET/CT should preferably be done prior to BMB, but this was not the case in six of 20 patients. However, previous work has shown that performing BMB before FDG-PET/CT does not result in any visually increased FDG uptake that is regarded as pathological [11]. Furthermore, BMB-induced anatomical CT changes at the posterior iliac crest are very uncommon in patients who undergo BMB before FDG-PET/CT, and, if present, have a characteristic millimetric cylindrical shape that is not to be confused with pathology [11]. Thus, it is unlikely that BMB has affected FDG-PET/CT interpretation in these six patients. Fourth, FDG-avid bone lesions outside of the posterior iliac crest were not biopsied, as mentioned previously. Fifth, FDG-PET/CT was not compared to bone scintigraphy or MRI. One previous study by Ulaner et al. [12] compared FDG-PET/CT to bone scintigraphy in 60 newly diagnosed Ewing sarcoma patients, however without reporting their diagnostic values compared to BMB. In their study, 40 primary malignancies had a lytic CT appearance, three were sclerotic, and 13 involved only soft tissue [12]. In 11 of 12 patients with bone marrow metastases, these were detected on FDG-PET/CT, with the one false-negative occurring in a sclerotic primary tumor [12]. In nine of 12 patients with bone marrow metastases, these were detected on bone scintigraphy, with the three false-negatives occurring in patients with lytic primary tumors [12]. Ulaner et al. [12] concluded that when Ewing sarcoma is lytic, bone scintigraphy does not add to staging performed by FDG PET/CT, and may be omitted. However, in the uncommon situation of primary sclerotic Ewing sarcoma of bone, Ulaner et al. [12] stated that bone scintigraphy may detect osseous metastases not detected by FDG-PET/CT. The additional value of bone scintigraphy to FDG-PET/CT in primary extraosseous Ewing sarcoma is still unclear [12]. In the present study, one patient had non-FDG-avid BMB-proven bone involvement of the right posterior iliac crest that appeared sclerotic on corresponding low-dose CT (Fig. 2). This example demonstrates that both the PET and CT portion of the examination should always be reviewed to detect bone marrow metastases in Ewing sarcoma. Unlike bone scintigraphy that only demonstrates osteoblastic activity, both FDG-PET and MRI are also able to assess the bone marrow, similar to BMB. There are no studies that have directly compared FDG-PET/CT to (whole-body) MRI and BMB in newly diagnosed Ewing sarcoma. However, it should be noted that whole-body MRI is still time-consuming, and MRI is less sensitive than CT for detecting lung metastasis. Therefore, at present, FDG-PET/CT seems to be the primary whole-body staging method of choice in Ewing sarcoma.

In conclusion, FDG-PET/CT appears to be a valuable method for metastatic bone marrow assessment in newly diagnosed Ewing sarcoma. The routine use of blind BMB of the posterior iliac crest should be reconsidered when FDG-PET/CT is available.

## References

[1] Gaspar N, Hawkins DS, Dirksen U (2015). Ewing sarcoma: current management and future approaches through collaboration. J Clin Oncol.

[2] Esiashvili N, Goodman M, Marcus RB (2008). Changes in incidence and survival of Ewing sarcoma patients over the past 3 decades: surveillance epidemiology and end results data. J Pediatr Hematol Oncol.

[3] Bain BJ (2005). Bone marrow biopsy morbidity: review of 2003. J Clin Pathol.

[4] Harrison DJ, Parisi MT, Shulkin BL (2017). The role of 18F-FDG-PET/CT in pediatric sarcoma. Semin Nucl Med.

[5] Boellaard R, Delgado-Bolton R, Oyen WJ (2015). European Association of Nuclear Medicine (EANM). FDG PET/CT: EANM procedure guidelines for tumour imaging: version 2.0. Eur J Nucl Med Mol Imaging.

[6] Ladenstein R, Pötschger U, Le Deley MC (2010). Primary disseminated multifocal Ewing sarcoma: results of the euro-EWING 99 trial. J Clin Oncol.

[7] Biermann JS, Chow W, Reed DR (2017). NCCN guidelines insights: bone cancer, version 2.2017. J Natl Compr Cancer Netw.

[8] ESMO/European Sarcoma Network Working Group (2014). Bone sarcomas: ESMO clinical practice guidelines for diagnosis, treatment and follow-up. Ann Oncol.

[9] http://www.euroewing.eu/clinical-trials/ee2012-trial. Accessed 12 Aug 2017.

[10] Newman EN, Jones RL, Hawkins DS (2013). An evaluation of [F-18]-fluorodeoxy-d-glucose positron emission tomography, bone scan, and bone marrow aspiration/biopsy as staging investigations in Ewing sarcoma. Pediatr Blood Cancer.

[11] Adams HJ, de Klerk JM, Fijnheer R, Dubois SV, Nievelstein RA, Kwee TC (2016). Effect of blind bone marrow biopsy on FDG-PET/CT interpretation of the posterior iliac crest in diffuse large B-cell lymphoma. Hematol Oncol.

[12] Ulaner GA, Magnan H, Healey JH, Weber WA, Meyers PA (2014). Is methylene diphosphonate bone scan necessary for initial staging of Ewing sarcoma if 18F-FDG PET/CT is performed?. AJR Am J Roentgenol.

